# An Audit on the Management of Adult Patients With Renal Colic Presenting to the Emergency Department

**DOI:** 10.7759/cureus.118507

**Published:** 2026-10-24

**Authors:** Heba M Ahmed, Sara E Almheiri, Esra M Aldarmaki, Reem S Al Thehli, Khawla S Abdulrahman, Shahla K Al Marrawi, Neena A Roy, Tamadur A El Shikhidris, Maha E Aljaberi, Maryam Al Nuaimi, Nasser Ahmed, Kenneth C Dittrich, Ismail Mohamed Al Ramahi, Hasan Qayyum

**Affiliations:** 1 Emergency Department, Sheikh Khalifa Medical City, Abu Dhabi, ARE

**Keywords:** clinical audit, emergency department, nephrolithiasis, quality improvement, renal colic, ureteric colic

## Abstract

Introduction: Renal colic is a common emergency department presentation requiring timely assessment, appropriate analgesia, and investigation. This clinical audit evaluated adherence to predefined standards for pain assessment, timely analgesia, investigations, imaging, and medication selection, and assessed changes in practice following a structured implementation phase.

Methods: A retrospective, single-center, two-cycle clinical audit was conducted in an adult emergency department. The primary audit included 100 patients presenting between December 2020 and December 2024, and the re-audit included 100 patients presenting between March and September 2025, giving a total sample of 200 patients. Nine audit criteria were derived from the Royal College of Emergency Medicine (RCEM) and the National Institute for Health and Care Excellence (NICE) guidance. Following the primary audit, departmental education, audit feedback, an educational infographic, and a standardized renal colic analgesia pathway were implemented. Categorical outcomes were reported as frequencies and percentages and compared between audit cycles using two-sided Chi-square or Fisher exact tests, as appropriate.

Results: First-line nonsteroidal anti-inflammatory drug (NSAID) administration increased from 39/100 patients (39.0%) in the primary audit to 60/100 patients (60.0%) in the re-audit, representing an absolute increase of 21.0 percentage points (95% CI: 7.5-34.5). This difference was statistically significant (χ²(1, N = 200) = 8.00; p = 0.005). Avoidance of first-line opioid administration increased from 89/100 patients (89.0%) to 94/100 patients (94.0%); however, this difference was not statistically significant (χ²(1, N = 200) = 1.03; p = 0.310). Pain-score documentation, timely analgesia, urine dipstick testing, laboratory investigations, computed tomography (CT) imaging, and avoidance of antispasmodic medication remained broadly consistent between the two audit cycles.

Conclusion: Following implementation of a standardized renal colic pathway, departmental education, an educational infographic, and audit feedback, first-line NSAID use increased significantly and first-line opioid use decreased. Other key measures of assessment, investigation, and analgesic care remained generally stable between audit cycles. These findings support the value of a structured departmental approach to promoting guideline-concordant analgesic prescribing and provide a basis for continued quality-improvement activity and future re-audit.

## Introduction

Renal colic is a common presentation in emergency departments, yet clinical workup and management vary. The Royal College of Emergency Medicine (RCEM) clinical audit standards specify documentation of pain scores, timely analgesia, urinalysis, blood testing, and consideration of radiological investigation [1]. The National Institute for Health and Care Excellence (NICE) recommends a nonsteroidal anti-inflammatory drug (NSAID) as first-line analgesia, intravenous paracetamol when NSAIDs are contraindicated or ineffective, opioids only when both are contraindicated or ineffective, and avoidance of antispasmodics [2].

Renal colic is a common emergency department presentation caused most frequently by acute ureteric obstruction. The reported prevalence of urinary stone disease varies geographically and has increased over time in several populations [3-7]. In the United States, kidney stone prevalence and emergency presentations have increased, with associated admissions and higher healthcare expenditure [8-12]. Male sex, metabolic disease, obesity, dietary factors, and reduced fluid intake are recognized epidemiological associations [3-7,13].

Clinical management varies according to pain severity, evidence of infection or obstruction, renal function, pregnancy status, and the probability of spontaneous stone passage. Emergency assessment generally includes pain scoring, urinalysis, renal function testing, and appropriate imaging. Noncontrast computed tomography (CT) is the gold standard reference for most nonpregnant adults, while ultrasound is preferred when ionizing radiation should be avoided [14-16].

This audit aimed to describe the demographic and clinical characteristics of adults presenting with renal colic and to measure emergency department practice against nine quality standards defined a priori. A re-audit was performed after departmental education, utilization of infographics, and the introduction of a standardized renal colic analgesia pathway.

## Materials and methods

Design and setting

This was a retrospective, single-center, two-cycle clinical audit undertaken in the adult emergency department at Sheikh Khalifa Medical City, Abu Dhabi, United Arab Emirates. The audit proposal was submitted to the institutional Quality Department before data collection. As the project was conducted as a service evaluation, formal research ethics approval was not required.

Participants and data collection

The primary audit used a convenience sample of 100 adults aged 16 years or older who presented between December 2020 and December 2024 with a recorded diagnosis of renal colic, ureteric colic, renal stone, ureteric stone, or urinary stone. The re-audit used the same diagnostic inclusion criteria and included 100 patients presenting between March and September 2025. Data were obtained from the electronic health record and entered into Microsoft Excel (Microsoft Corporation, Redmond, Washington, United States). Data were collected by a team of emergency medicine residents who were supervised by an emergency department consultant. Patient identifiers were removed before analysis.

Audit standards

Nine locally agreed audit criteria were developed by the authors from the RCEM renal colic audit framework (criteria 1-5) and NICE guideline NG118 (criteria 6-9) [3,4]. The author-generated summary of criteria and targets is shown in Table 1.

**Table 1:** Audit criteria and standards from the RCEM renal colic audit standards and NICE NG118 recommendations ED: emergency department; RCEM: Royal College of Emergency Medicine; NICE: National Institute for Health and Care Excellence; CT: computed tomography; NSAID: nonsteroidal anti-inflammatory drug Source: [1,2]

Criterion	Author-generated audit criterion	Target
1	Record a pain score during the initial ED assessment^1^	100%
2	For severe pain (score 7-10), provide appropriate analgesia within 60 minutes of arrival or triage^1^	98%
3	For moderate pain (score 4-6), provide appropriate analgesia within 60 minutes of arrival or triage^1^	90%
4	Perform bedside urine dipstick testing^1^	100%
5	Obtain complete blood count and renal function testing^1^	100%
6	Use noncontrast CT when clinically applicable and not contraindicated^2^	100%
7	Use an NSAID as initial analgesic therapy unless contraindicated^2^	100%
8	Avoid opioids as initial therapy; reserve them for cases in which NSAIDs and paracetamol are contraindicated or ineffective^2^	100%
9	Avoid routine antispasmodic therapy^2^	100%

Implementation phase

The implementation phase was conducted from January to March 2025. The primary audit findings were presented at a departmental meeting and summarized in the departmental governance newsletter. A renal colic infographic (Figure 1) was displayed as an aide-mémoire, and a pain pathway (Figure 2) was introduced specifying NSAIDs as first-line therapy, followed by intravenous paracetamol and then opioid analgesia when preceding options were contraindicated or ineffective. Departmental communications also addressed pain-score documentation, urine dipstick testing, blood investigations, CT imaging, and avoidance of routine antispasmodic use.

**Figure 1:**
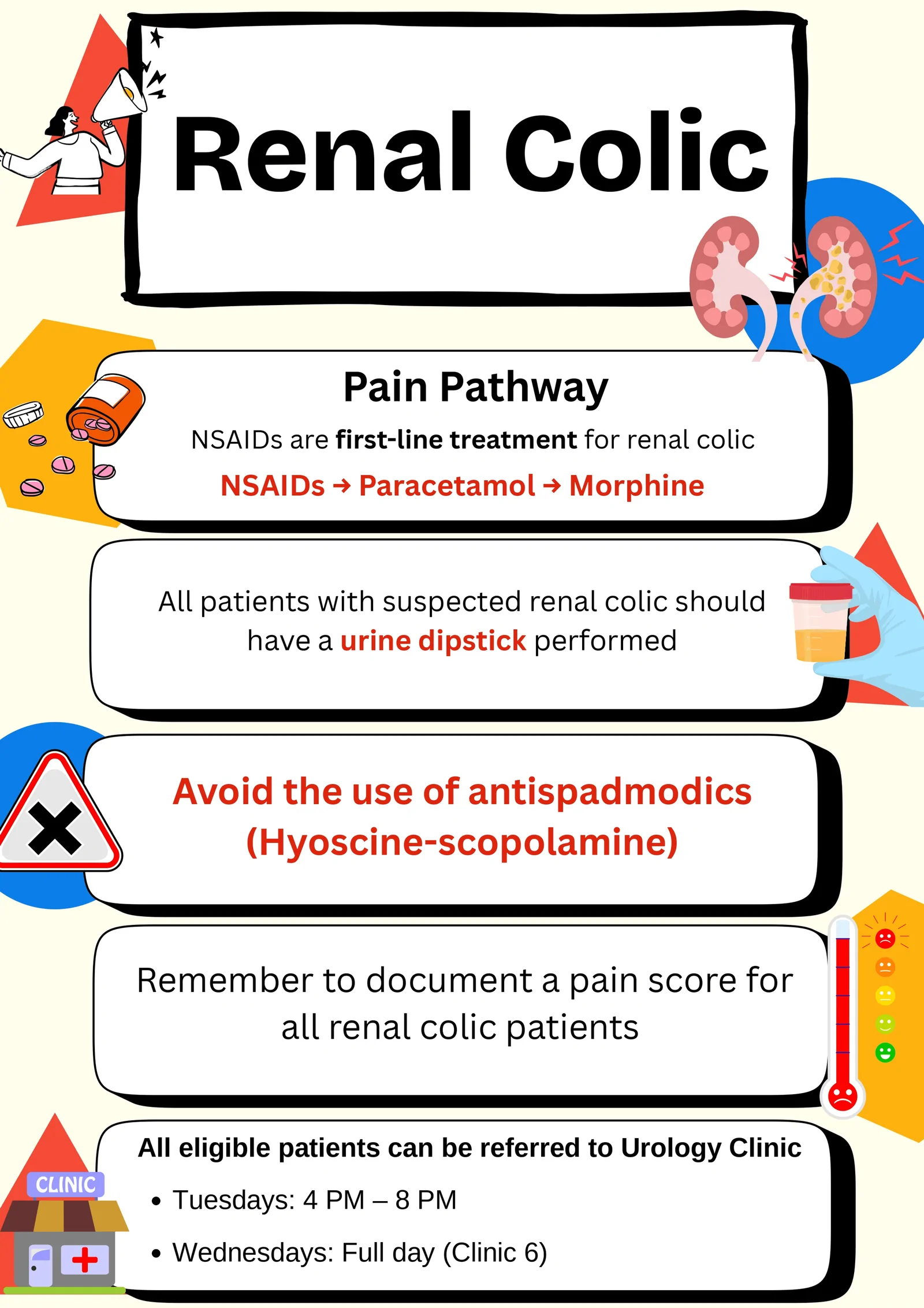
Renal colic infographic shared with our emergency department as an aide memoire Image credit: Created by Qayyum H using Canva (Canva Inc., Perth, Australia)

**Figure 2:**
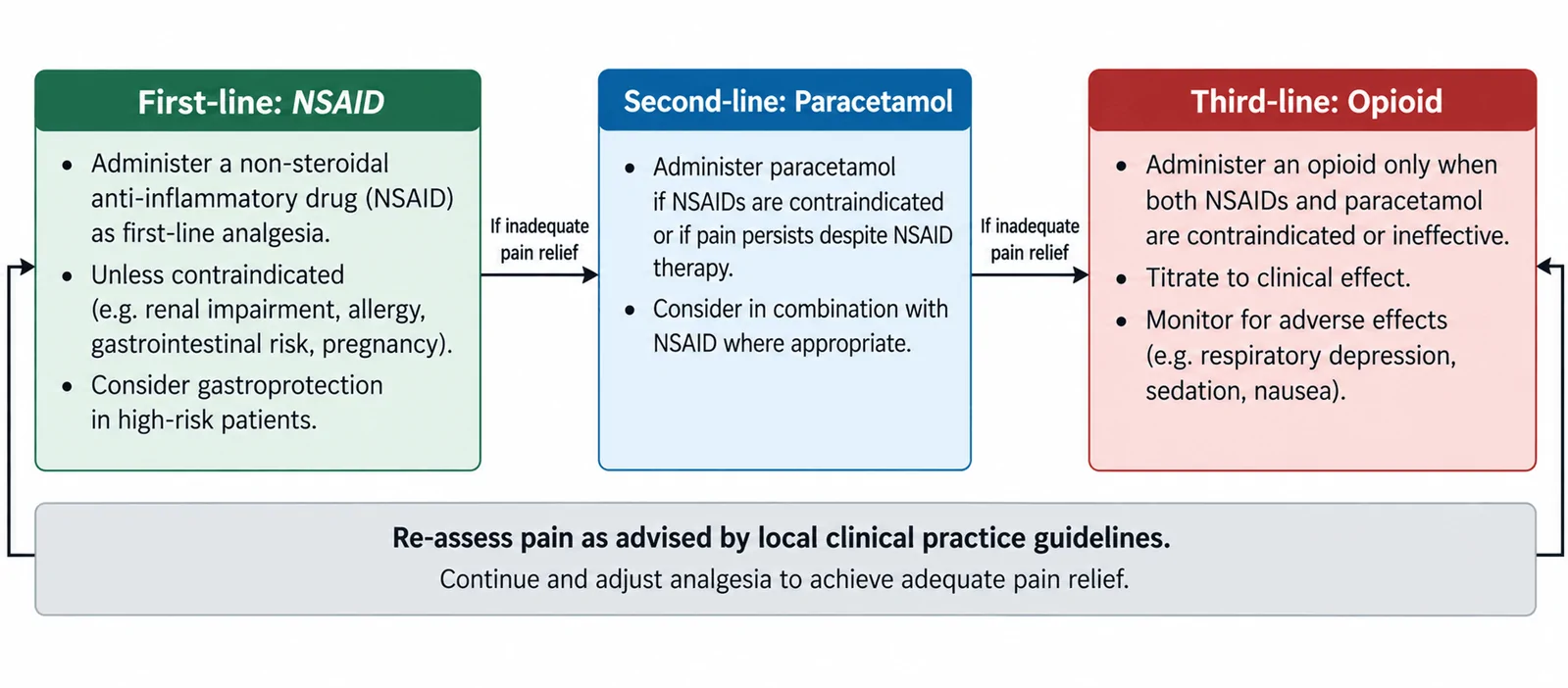
Renal colic pain pathway highlighting importance of first line nonsteroidal anti-inflammatory use Image credit: Created by Qayyum H using Canva (Canva Inc., Perth, Australia)

Categorical outcomes were reported as frequencies (n) and percentages. Differences between the primary audit and re-audit were expressed as absolute percentage-point differences with 95% confidence intervals (CIs). Two-sided Pearson Chi-square tests were used when expected cell counts were adequate; Fisher exact tests were used for sparse cells. For Pearson Chi-square tests, the test statistic, degrees of freedom, and phi coefficient (phi; equivalent to Cramer's V for 2 x 2 tables) were reported. A p-value below 0.05 was considered statistically significant. Statistical comparisons were exploratory and were not adjusted for multiple testing.

## Results

Study population and demographics

A total of 200 patients were included: 100 in the primary audit and 100 in the re-audit. The primary audit cohort included 73 men (73%) and 27 women (27%), with a mean age of 43 years. Thirty-nine patients (39%) presented between 06:00 and 14:00, forty patients (40%) between 14:00 and 22:00, and twenty-one (21%) between 22:00 and 06:00. Seventy-seven patients (77%) were assigned Emergency Severity Index (ESI) category 3 and 23 (23%) were assigned ESI category 2 [17]. Corresponding demographic and triage variables were not available in the re-audit summary dataset.

Primary audit

Pain scores were documented in all 100 patients (100%). Of the 37 patients presenting with severe pain, 36 (97.3%) received appropriate analgesia within 60 minutes. Of the 63 patients with moderate pain, 55 (87.3%) received appropriate analgesia within 60 minutes. Urine dipstick testing was performed in 85 patients (85.0%). Complete blood count and renal function tests were performed in all 100 patients (100%), and noncontrast CT was performed in 94 patients (94.0%).

An NSAID was administered as first-line analgesia in 39 patients (39.0%). Twenty-seven patients (27.0%) required opioid analgesia following NSAIDs and/or intravenous paracetamol, whereas 62 patients (62.0%) did not require opioid escalation. Eleven patients (11.0%) received an opioid as first-line analgesia; consequently, 89 patients (89.0%) met the criterion for avoidance of first-line opioid use. Antispasmodic medication was administered to 21 patients (21.0%), with 79 patients (79.0%) meeting the avoidance criterion.

Re-audit

Pain scores were documented in 97 patients (97.0%). Of the 28 patients presenting with severe pain, 25 (89.3%) received appropriate analgesia within 60 minutes. Of the 72 patients with moderate pain, 61 (84.7%) received appropriate analgesia within 60 minutes. Urine dipstick testing was performed in 84 patients (84.0%). Complete blood count and renal function tests were performed in all 100 patients (100%). Noncontrast CT was performed in 95 patients (95.0%); in the remaining five patients, contrast-enhanced CT was performed to investigate an alternative diagnosis.

An NSAID was administered as first-line analgesia in 60 patients (60.0%). Opioid escalation was required when there was an inadequate response to paracetamol and/or NSAIDs in eight patients. Six patients (6.0%) received first-line opioids, and 94/100 patients (94.0%) met the avoidance criterion. Antispasmodics were administered to 21/100 patients (21.0%); 79/100 patients (79.0%) met the avoidance criterion.

Comparison between audit cycles

The most notable improvement between audit cycles was the increase in first-line NSAID administration from 39.0% to 60.0%, representing an absolute increase of 21.0 percentage points (95% CI: 7.5-34.5; p = 0.005) (Figure 3). 

**Figure 3:**
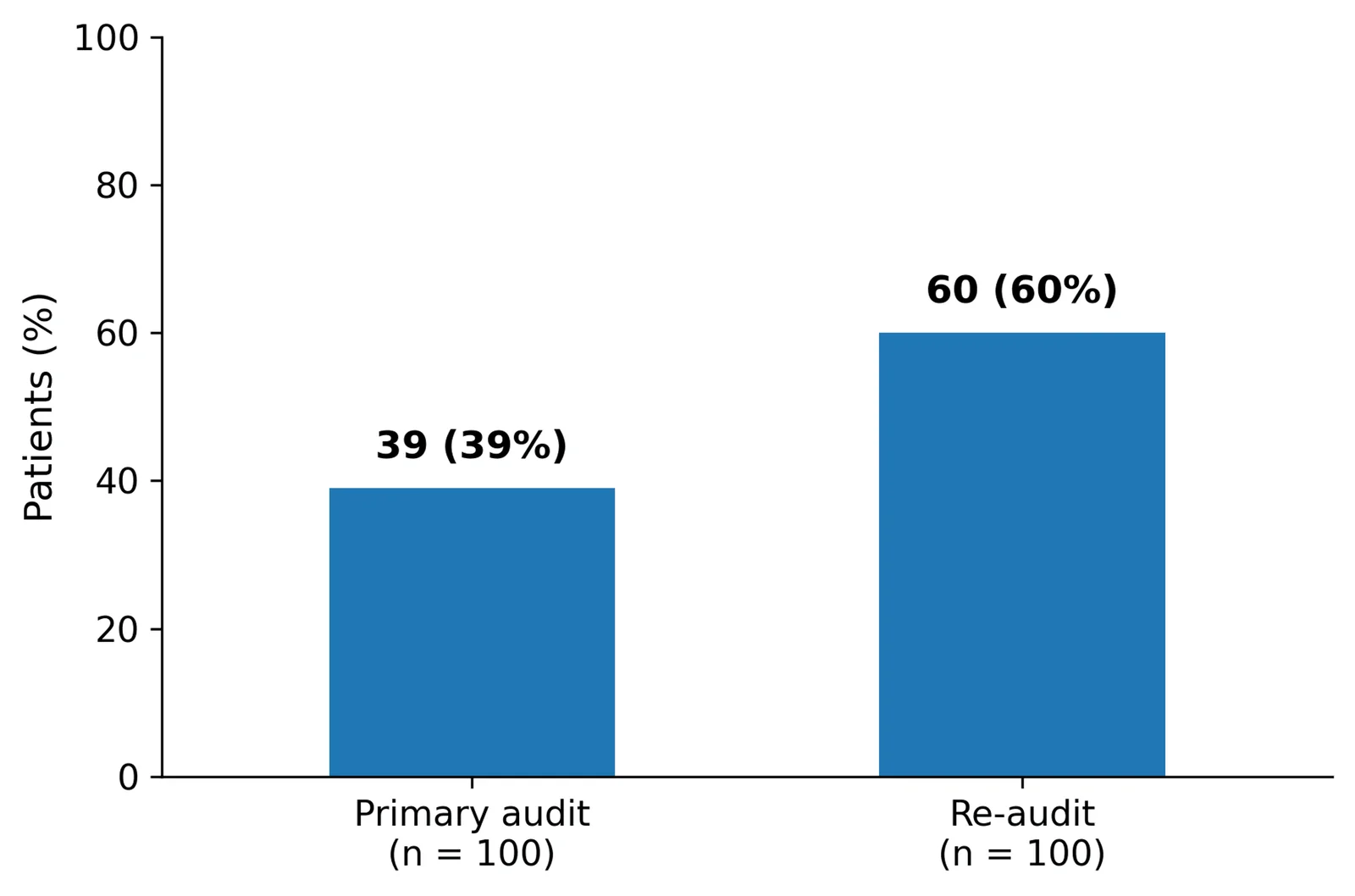
First line NSAID use in the primary audit versus the re-audit NSAID: nonsteroidal anti-inflammatory drug

Changes in opioid prescribing and escalation are shown in Figure 4. The proportion of patients requiring opioid escalation following NSAIDs and/or intravenous paracetamol decreased from 27 patients (27.0%) to eight patients (8.0%), while the proportion not requiring opioid escalation increased from 62 patients (62.0%) to 86 patients (86.0%). First-line opioid administration also decreased from 11 patients (11.0%) to six patients (6.0%), corresponding to an increase in avoidance of first-line opioid use from 89.0% to 94.0%; however, this difference was not statistically significant (p = 0.311).

**Figure 4:**
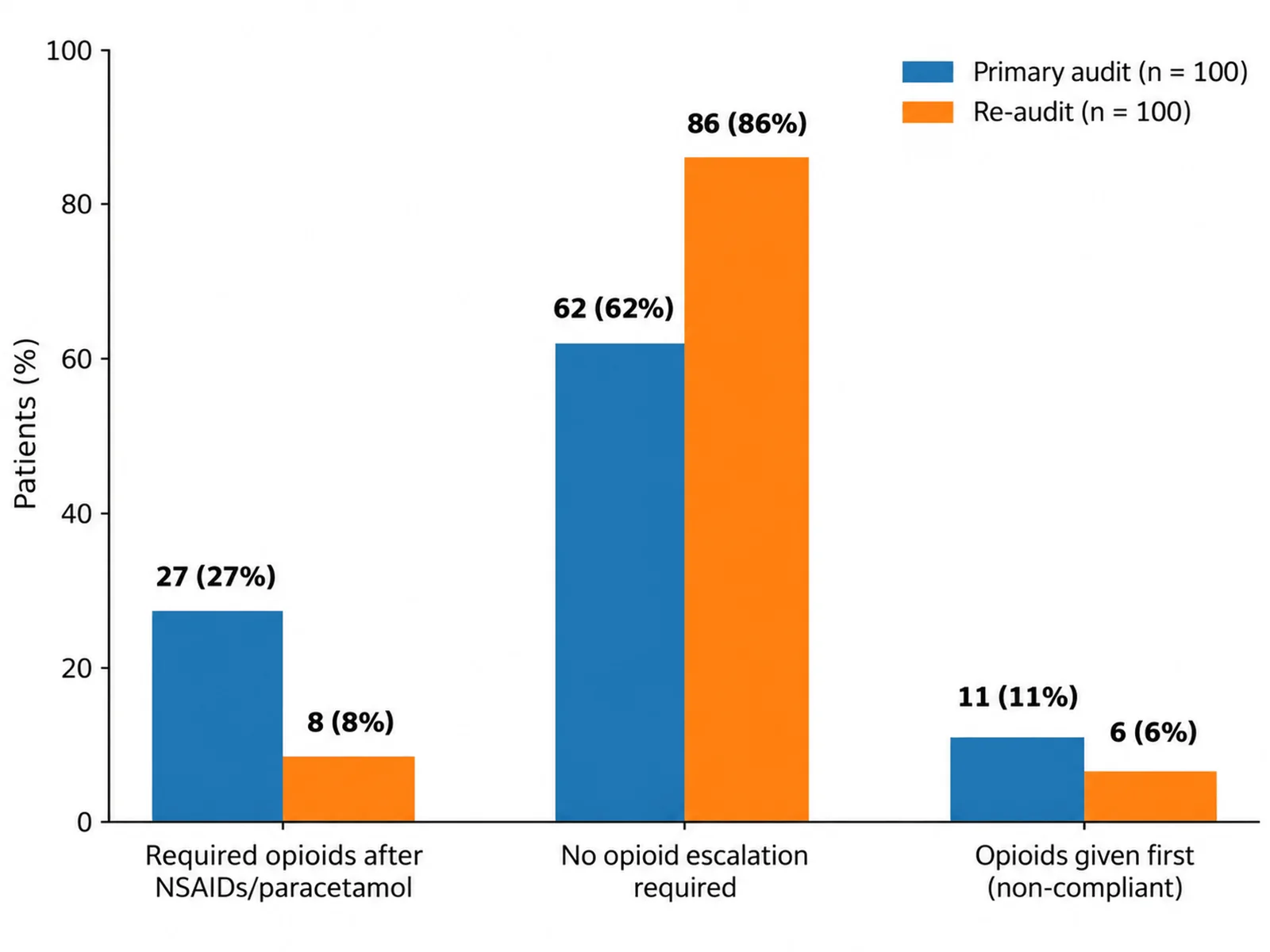
Opioid prescribing practices in the primary audit and re-audit NSAID: nonsteroidal anti-inflammatory drug

The audit criteria results for both the primary audit and the re-audit are elaborated in Table 2.

**Table 2:** Comparison of audit criteria for the primary audit and the re-audit NSAID: nonsteroidal anti-inflammatory drug; CT: computed tomography; CBC: complete blood count

Criteria	Primary audit, n/N (%)	Re-audit, n/N (%)
Pain score documented	100/100 (100.0)	97/100 (97.0)
Severe pain: analgesia within 60 min	36/37 (97.3)	25/28 (89.3)
Moderate pain: analgesia within 60 min	55/63 (87.3)	61/72 (84.7)
Urine dipstick performed	85/100 (85.0)	84/100 (84.0)
CBC and renal function tests	100/100 (100.0)	100/100 (100.0)
Noncontrast CT performed	94/100 (94.0)	95/100 (95.0)
NSAID as first-line analgesia	39/100 (39.0)	60/100 (60.0)
Avoidance of first-line opioid	89/100 (89.0)	94/100 (94.0)
Avoidance of antispasmodics	79/100 (79.0)	79/100 (79.0)

## Discussion

This audit identified high baseline compliance with pain-score documentation, blood investigations, and CT imaging. After implementing audit feedback, education, an infographic, and a renal colic analgesia pathway, first-line NSAID use increased by 21.0 percentage points. Beyond statistical significance, this finding is clinically meaningful, as it indicates that 21 additional patients per 100 presentations received guideline-recommended first-line analgesia in the re-audit compared with the primary audit. NSAIDs are particularly appropriate for renal colic because they reduce prostaglandin-mediated renal pelvic pressure and ureteric edema, thereby targeting important mechanisms underlying renal colic pain. The observed improvement suggests a positive shift toward evidence-based analgesic prescribing following departmental education and implementation of a standardized renal colic pathway.

Nevertheless, first-line NSAID use remained at 60%, indicating further opportunities to optimize prescribing practices. Importantly, NSAID contraindications were not separately accounted for; therefore, the observed prescribing rate should not be interpreted as a definitive measure of guideline adherence. Furthermore, the audit did not directly assess improvements in pain relief or other patient-centered outcomes. Timely analgesia, urine dipstick testing, and avoidance of antispasmodics did not change statistically. Furthermore, we acknowledge that although NSAID administration increased significantly, the overall prescribing rate remained at 60%, highlighting that adoption of change in clinical practice takes time and sustained effort, indicating opportunities for further improvement. It was also observed that avoidance of first-line opioid administration increased by 5.0 percentage points.

Antispasmodic use remained at 21% across both audit cycles, highlighting an opportunity for further quality improvement. While the initial interventions did not change prescribing practice in this area, the findings provide a clear focus for targeted education, clinician feedback, and future re-auditing to improve adherence to guidelines. Continued prescribing may reflect established clinical practice or individual prescribing preferences, although these factors were not formally assessed.

The limited improvement in timely analgesia, urine dipstick testing, and antispasmodic avoidance may reflect competing clinical priorities, variations in practice, and established prescribing habits. Although these factors were not specifically assessed, the findings suggest that education and clinical pathways alone may be insufficient to change practice. Further targeted interventions and regular re-auditing may help improve adherence to guidelines.

Urinary stone disease is common and has increased in prevalence over recent decades [3-7]. Population studies have described substantial emergency department utilization, hospital admissions, procedural intervention, and healthcare expenditure [8-12]. The male predominance in the primary audit was consistent with historical epidemiological reports, although sex differences have narrowed in some contemporary populations [3-7,13]. In the Middle East, climatic conditions, dehydration, diet, and regional patterns of calcium oxalate and uric acid stones contribute to disease burden [18].

Renal colic results from acute obstruction, increased intraluminal pressure, ureteric distension, edema, and prostaglandin-mediated changes in renal blood flow [19-21]. Symptoms typically include unilateral flank pain, nausea or vomiting, and microscopic or macroscopic hematuria [13,22]. Hematuria supports but does not establish the diagnosis: studies have shown that urinary calculi may occur without hematuria and that hematuria may be present without a stone on imaging [23-25]. Urinalysis also assists in identifying pyuria, nitrites, or leucocyte esterase that may indicate concomitant urinary tract infection [26]. An infected obstructed collecting system requires urgent urological assessment and decompression.

Complete blood count and renal function testing were performed in all patients during both cycles. These tests support assessment for infection, acute kidney injury, and complications of obstruction, although results require interpretation alongside the clinical presentation [13,19-21]. Urine dipstick testing remained incomplete in both cycles, at 85.0% and 84.0%, despite its inclusion in the pathway and audit criteria.

Noncontrast CT was performed in 94 patients (94.0%) in the primary audit and 95 patients (95.0%) in the re-audit. CT has high diagnostic accuracy for ureteric calculi and can identify stone size, location, obstruction, and alternative diagnoses [27,28]. Plain radiography has lower diagnostic sensitivity and is limited by radiolucent stones and overlying structures [28]. Ultrasound can identify hydronephrosis and some renal or ureteric stones, but obstruction may occur without hydronephrosis [29-31]. The color Doppler twinkling artifact and assessment of ureteric jets can support sonographic diagnosis [32-35]. Ultrasound avoids ionizing radiation and is therefore particularly relevant during pregnancy [36,37], while broader reviews describe complementary roles for CT, ultrasound, and radiography according to patient characteristics and clinical context [38].

The main post-implementation change concerned analgesic selection. NSAIDs reduce prostaglandin-mediated renal pelvic pressure and ureteric edema and are recommended as first-line treatment by NICE and European guidance [2,14]. The increase from 39 patients (39.0%) to 60 patients (60.0%) remained below the audit target of 100%, but represented a statistically significant change. First-line opioid administration decreased from 11 patients (11.0%) to six patients (6.0%). Opioids remain an option when NSAIDs and intravenous paracetamol are contraindicated or ineffective, but routine first-line use is not recommended [2,13,14].

Antispasmodic avoidance was 79.0% in both the primary audit and re-audit. NICE guidance advises against antispasmodics for suspected renal colic [2]. The absence of change indicates that this component of the pathway was not reflected in the measured prescribing outcome during the re-audit period.

Stone size and location influence spontaneous passage and the need for intervention. Alpha-blockers have been evaluated as medical expulsive therapy, with systematic reviews reporting the greatest potential benefit for larger distal ureteric stones [39,40]. Clinical prediction rules such as the STONE score have also been externally validated as aids to estimating the probability of ureteric stone, although they do not replace clinical assessment or imaging when alternative diagnoses or complications are possible [41]. Definitive management may include shock-wave lithotripsy, ureteroscopy, or percutaneous nephrolithotomy, depending on stone characteristics and patient factors [14,42]. Pregnancy requires modified diagnostic and management strategies to minimize maternal and fetal risk [36,37,43].

The audit used predefined criteria derived from national guidance and repeated measurement after a defined implementation phase. Limitations include its retrospective single-center design, convenience sampling, dependence on documentation in the electronic record, and absence of patient-centered outcomes such as change in pain score, length of stay, adverse effects, return attendance, stone passage, admission, or intervention. The nonrandomized study design and the absence of adjustment for differences in patient case mix limit the ability to attribute observed changes directly to the intervention. The relatively small sample size and short re-audit period may also limit generalizability. The primary audit covered a longer sampling period than the re-audit, and demographic variables were not available in the re-audit summary dataset. The absence of re-audit demographic and triage data was also a limitation when comparing the two audit cycles. The study therefore measured short-term changes in selected processes of care and did not assess long-term sustainability. 

## Conclusions

Implementation of a standardized renal colic pathway, departmental education, an infographic, and audit feedback were followed by increased first-line NSAID administration and reduced first-line opioid administration. Pain-score documentation, blood investigations, and CT imaging remained high in both cycles. Timely analgesia for moderate pain, urine dipstick testing, and avoidance of antispasmodics did not change statistically and remained below their predefined targets. Further audits and quality improvement exercises should assess whether the observed prescribing changes are sustained and should include patient-centered outcomes, reasons for nonadherence, contraindications to NSAIDs, and balancing measures such as adverse drug effects and unplanned emergency department return attendances.
